# Glenoid Component Loosening in Anatomic Total Shoulder Arthroplasty: Association between Radiological Predictors and Clinical Parameters—An Observational Study

**DOI:** 10.3390/jcm10020234

**Published:** 2021-01-11

**Authors:** Alexandra Grob, Florian Freislederer, Alex Marzel, Laurent Audigé, Hans-Kaspar Schwyzer, Markus Scheibel

**Affiliations:** 1 Shoulder and Elbow Surgery, Schulthess Clinic, 8008 Zurich, Switzerland; Alexandra.Grob@triemli.zuerich.ch (A.G.); Florian.Freislederer@kws.ch (F.F.); Alex.Marzel@kws.ch (A.M.); Laurent.Audige@kws.ch (L.A.); Hans-Kaspar.Schwyzer@kws.ch (H.-K.S.); 2 Teaching, Research and Development Department, Schulthess Clinic, 8008 Zurich, Switzerland; 3 Center for Musculoskeletal Surgery, Charité-Universitaetsmedizin, 10117 Berlin, Germany

**Keywords:** total shoulder arthroplasty (TSA), glenoid component loosening, risk factors, radiography, pegged glenoid component

## Abstract

The mechanisms of glenoid component loosening in anatomic total shoulder arthroplasty (aTSA) are still unclear, and it remains undetermined which specific radiographic features are associated with clinical outcomes. Patients with primary osteoarthritis who underwent aTSA with a stemless implant and a pegged glenoid between January 2011 and December 2016 were extracted from a local registry. Anteroposterior radiographs were evaluated at six, 12, 24 months, and five years post-TSA for lateral humeral offset (LHO), joint gap (JG), acromiohumeral distance (AHD), and radiolucency (modified Franklin score); 147 patients were included. Mixed-model linear regression was used. Both constant score (CS) and subjective shoulder value (SSV) markedly decreased at five years follow-up compared to one year (*p* < 0.001 for both). AHD, LHO, and JG all showed a consistent and statistically significant decline over time, with the joint gap decreasing by half. Consistently, smaller JG and AHD were correlated with lower SSV (*p* = 0.03 and *p* = 0.07, respectively). Massive loosening was associated with a 14.5 points lower SSV (*p* < 0.01). Finally, narrowing of the JG was significantly correlated with increased radiolucency (*p* < 0.001) and tended toward worse SSV (*p* = 0.06). In summary, radiographic parameters displaying medialization and cranialization after aTSA with a cemented pegged glenoid are useful predictors of impaired shoulder function.

## 1. Introduction

Anatomic total shoulder arthroplasty (aTSA) has been shown to be an effective treatment for primary glenohumeral arthritis [1,2,3,4,5], with good pain reduction and restoration of joint mobility. Glenoid component loosening in aTSA is a leading complication and a common reason for implant failure and revision surgery [6,7,8].

Patient-reported pain, along with clinical findings, such as active range of motion or strength, and radiographs with radiolucent lines are all important factors for the detection of implant loosening [1,2,4,9,10]. The role of radiolucency around the implants has been widely discussed, with the common finding of their prevalence and progression over time in cases of loosening [11,12,13]. Further, several publications showed that clear signs of radiolucency occurring shortly after surgery and their further progression are correlated with implant loosening [14,15,16]. Nevertheless, radiolucency can be very common after cemented polyethylene (PE) pegged glenoid component implantation, with a reported prevalence reaching, in some cases, 96% [17,18]. This minimizes its specificity as a screening parameter for future loosening and implant failure [19], and emphasizes the need for additional radiographic predictors [20]. Alongside with radiolucency as a sign for implant loosening, further radiographic observable indicators, like superior shift of the humeral head [21,22] and medialization due to erosion of the glenoid side [5], have been previously reported. However, data on the dynamics of the humeral head shift and its causal link to glenoid loosening post-arthroplasty is still very scarce. Most of the available investigations on implant failure mechanisms have been performed on keeled glenoids, and little is known about the dynamics of performance of the pegged glenoid components over time.

The purpose of this observational study and main outcome was to correlate the long-term longitudinal association of radiographic parameters with clinical findings, so that they can be used as an early indication for glenoid component loosening after anatomic TSA with the use of a cemented all PE pegged glenoid component for primary glenohumeral arthritis.

## 2. Materials and Methods

### 2.1. Inclusion Criteria and Follow-Up Time Points

Adult patients undergoing a stemless total shoulder arthroplasty with a pegged glenoid for primary osteoarthritis and registered in the local Shoulder Arthroplasty Registry (SAR) at the Schulthess Clinic, a high-volume orthopedic center in Zurich, Switzerland, were included. Patients were examined pre-operatively and at standardized follow-up time points at six, 12, 24 months and five years. This observational study was approved by the Ethical Committee of Canton Zürich (KEK-ZH-Nr. 2014-0483) and all patients provided consent.

### 2.2. Implant Description

In all cases, same cemented, all polyethylene, pegged glenoid component (Arthrex, Naples, FL, USA) in combination with a stemless implant on the humeral side (Eclipse, Arthrex, Naples, FL, USA) was used.

### 2.3. Surgical Technique and Postoperative Care

Surgical indication required severe shoulder pain and disability and the diagnosis of primary osteoarthritis with already exhausted conservative treatment. Most (77%) of the surgeries were performed by two experienced senior surgeons, and the rest were performed by specialized shoulder surgeons. The surgical technique of insertion of the PE pegged glenoid (Arthrex, Naples, FL, USA) has been previously described [23]. Standardized tenotomy was used for subscapularis release, and the tendon ends were reattached with a non-absorbable suture at the end of the surgery. Modifying washing process lavage was performed with a syringe to clean the bony glenoid surface from tissue debris. The glenoid surface was then dried with sterile dressings. Preparation of the glenoid involved guide pin placement at the position for the future central fixation hole. Cartilage was then removed with a reamer to build a concentric seating for the pegged component. The central fixation hole was drilled over the guide pin, and peripheral peg sites were drilled with the help of the template instrument. A fitting PE pegged glenoid size was chosen. Cement was inserted into the peg holes of the glenoid and pressed around the pegs. The PE glenoid component was inserted and held until the cement was cured.

Postoperatively, all patients wore a sling for six weeks. Internal rotation against resistance was to be avoided for six weeks while limited passive mobilization was carried out by physiotherapists. After 14 days, active mobilization was assisted in a limited range of motion. Active motion was progressively built up after one month. Functional training and strength exercises were supplemented when achieving a full range of motion.

### 2.4. Clinical and Radiological Evaluations

Clinical evaluation included the Constant–Murley Score (CS) [24], consisting of several subgroups of measurements representing pain, strength, range of motion, and impact on daily activities; the subjective shoulder value (SSV) [25], which is the shoulder function value, as estimated in percentage by the patient, with reference to a completely normal shoulder (100%).

**Figure 1:**
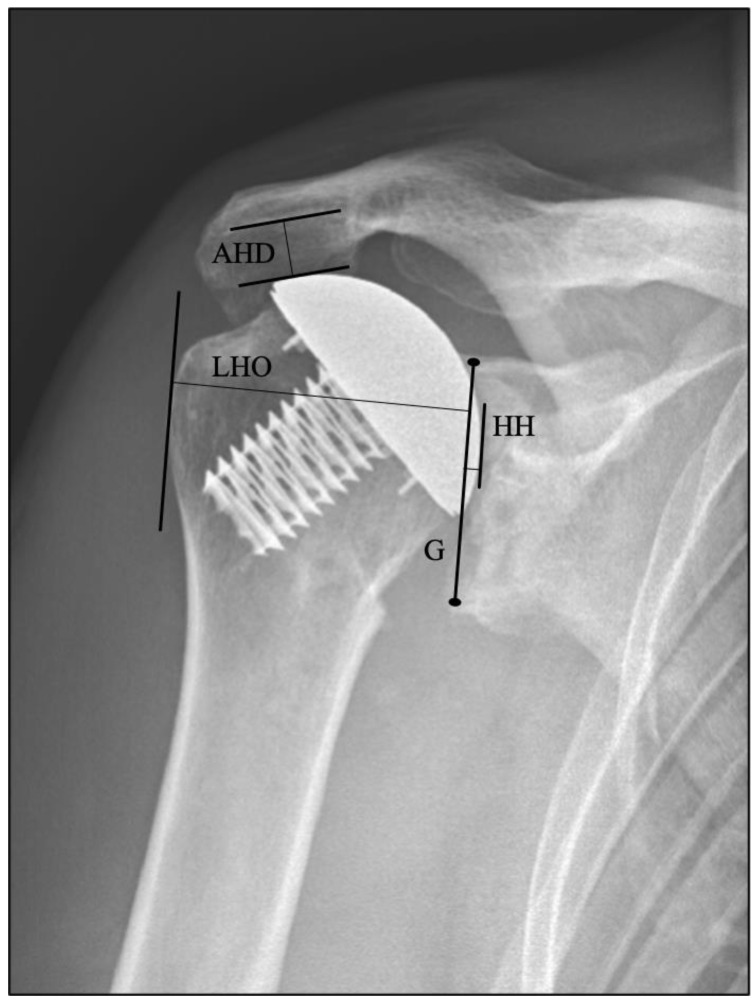
Anteroposterior measurements are illustrated on radiographs. The black line connects the superior and inferior rim of the glenoid (G) and is taken as the base for the joint gap width (black line to the right), which is the parallel line crossing the most medial part of the humeral head (HH) prosthesis. Positive values were given when the humeral head appeared more laterally relative to the drawn line, and negative values were given when more medially, respectively. The lateral humeral offset (LHO) is the distance (mm) measured from the drawn baseline to the parallel crossing the most lateral point of the greater tubercle. Acromiohumeral distance (AHD) is from the caudal point of the acromion perpendicular to the apex of the humeral head [28,29]. JG and LHO indicate a mediolateral shift of the humeral head, whereas the AHD represents cranialization.

True anteroposterior (AP) and axillary radiographs were obtained postoperatively at each follow-up examination according to a standardized internal protocol using a calibrated digital tool for imaging visualization (JiveX VISUS Health IT GmbH, Germany). All measurements were evaluated on AP views with internal rotation (Figure 1). To grade radiolucent lines, both radiographic projections, axillary and AP, were considered. Radiolucency was graded on a scale from 0–5 according to the extent of radiolucent lines at the bone-cement interface from Lazarus et al. [19], adjusted for pegged glenoids and originally implemented by Franklin et al. in 1988 for keeled glenoids [17]. The average of the grades from axillary and AP radiographs resulted in the final grading. To detect a mediolateral shift, the following parameters were measured: lateral humeral offset (LHO) and joint gap width (JG). Acromiohumeral distance (AHD) was evaluated to detect potential cranialization of the humeral head. For the assessment of craniocaudal positioning of the humeral head on plain radiographs, the AHD is a commonly used and reliable measurement [26]. Parameters of radiographs were measured by the same examiner, an orthopedic resident with experience in musculoskeletal radiology, blinded to the clinical outcomes. Glenoid morphology classification according to Walch was based on axillary radiographs, since not every patient had a CT scan preoperatively [27].

### 2.5. Statistical Analysis

The dataset was exported from the local SAR registry stored at the REDCap (Research Electronic Data Capture) server [30]. All statistical analysis was performed using R (v3.6.0, R Foundation for Statistical Computing, Vienna, Austria). Age-adjusted mixed-model linear regressions with per case random intercept (R package lme4) were applied to produce: (i) comparison of the least-square means of the examined radiographic parameters across time points, (ii) estimates of the association between radiographic parameters and CS and SSV across time points, and (iii) estimates of the association between the change in radiographic parameters (Δ from six months) and Franklin radiolucency grade, the CS and the SSV (at one, two, and five years).

## 3. Results

### 3.1. Patient Characteristics

**Figure 2:**
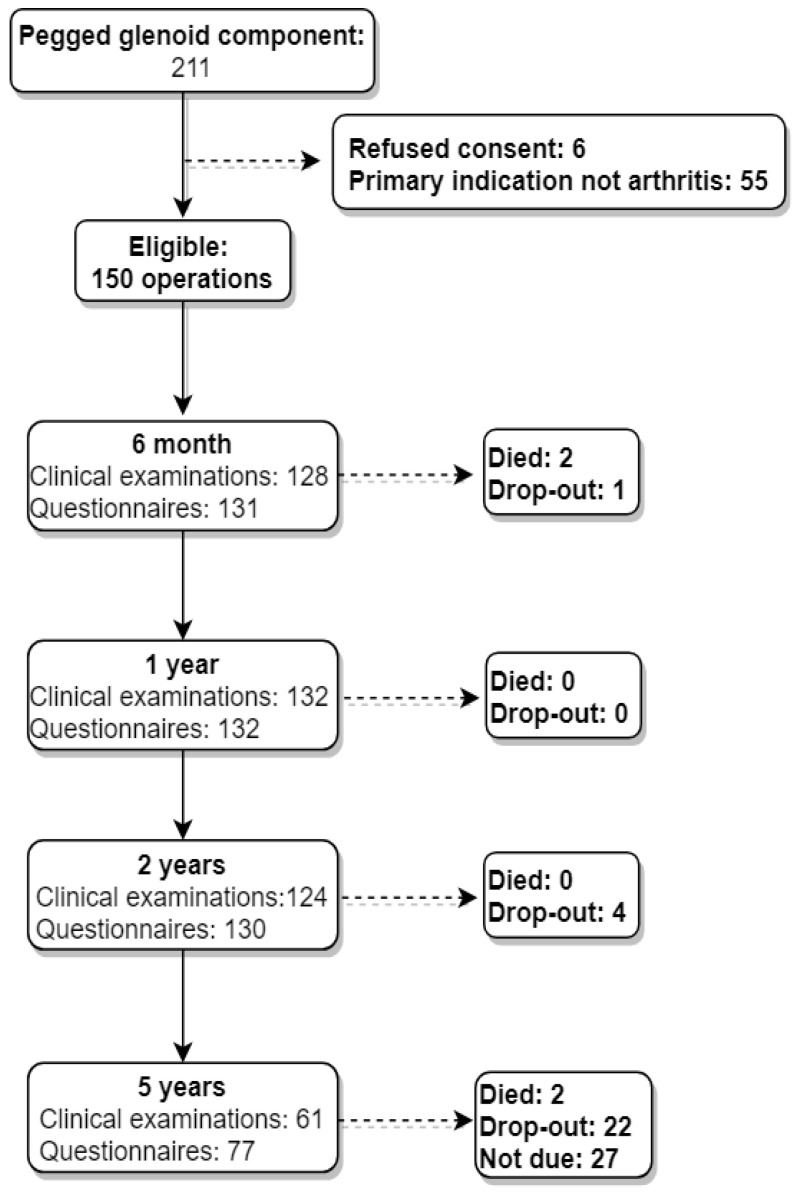
Study flow-chart.

Between January 2011 and December 2016, 211 cases of stemless aTSA with a pegged glenoid were enrolled in the SAR (Figure 2). Thereof, 156 cases were included with the diagnosis of primary osteoarthritis. Six patients were excluded due to rejected consent. At the end, 150 shoulders from 147 patients were included in the analysis. Mean age was 67 (±8, Table 1), 88 were women (59%), and 62 were men (41%). The majority received a small glenoid implant (*n* = 87, radius = 29 mm), 50 medium (radius = 30.5 mm), and 13 received a large glenoid component (radius = 32 mm). The most common humeral sizes were 43/16 (head radius = 23 mm) in 36 cases, 45/17 (head radius = 24.5 mm) in 31 cases, and 41/16 (head radius = 21.75 mm) in 27 cases.

### 3.2. Change in CS and SSV with Time

**Figure 3:**
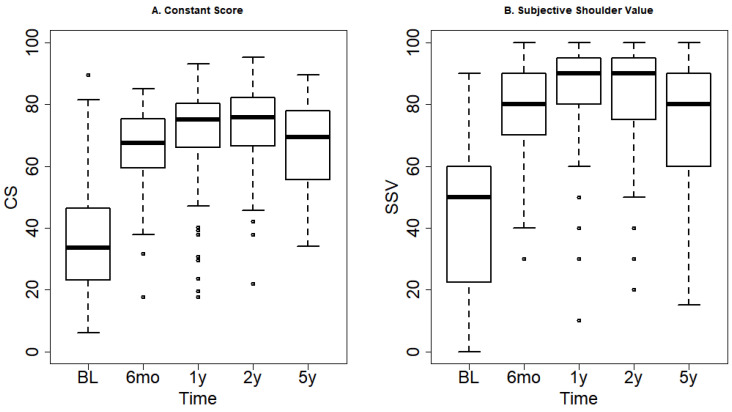
(**A**) Constant score (CS) and (**B**) Subjective shoulder value (SSV) over time. A large and clinically relevant decline is seen at 5 years follow-up for both outcomes. Abbreviations: BL, baseline; mo, months; y, year; CS, constant score; SSV, subjective shoulder value.

Post-operatively, CS and SSV improved to 71 and 83.5 at 12 months (*p* < 0.001 compared to baseline values, Figure 3), respectively. After a stable 24-month measurement, a simultaneous drop both in CS and SSV was observed at five years follow-up. Compared to the 12 month time point, CS declined by an average of 6.5 points (*p* < 0.001) and SSV by 8.9 points (*p* < 0.001) at five years follow-up.

### 3.3. Change in Radiographic Parameters with Time

The acromiohumeral distance, lateral humeral offset, and joint gap width all showed a consistent and statistically significant reduction over time (Table 2, *p* < 0.001 for the difference between five years vs. six months). The largest absolute declines occurred between the 24-month and five-year time points. The largest relative decline was for the joint gap, which was halved during the follow-up period (from 2.9 mm at six months to 1.4 mm at five years, *p* < 0.001).

### 3.4. Association between Radiographic Parameters and CS and SSV across Time Points

Over time, none of the radiographic parameters were significantly correlated with the constant score (Table 3). For the subjective shoulder value, both the joint gap and acromiohumeral distance showed a positive association (β = 1.51, *p* = 0.03, β = 0.50, *p* = 0.07, respectively). In contrast, Franklin grade 5 (gross loosening) showed a significant negative correlation with SSV that was associated with an average reduction of 14.5 percentage points (β = −14.5, *p* < 0.01), as compared to grade ≤ 1. The constant score and SSV define with accuracy and similar trajectory the outcome of various shoulder conditions [25]. The minimal clinically important difference (MCID) for the SSV has not been determined so far after anatomic shoulder arthroplasty for primary osteoarthritis. The MCID for the constant score after total shoulder arthroplasty is 5.7 [31]. A drop of 14.5 points for patients with gross loosening is a high drop in SSV, and, according to our experience, can be interpreted as a clinically significant difference.

### 3.5. Association between Change in Radiographic Parameters (Δ from Six Months) and Franklin Radiolucency Grade, the CS, and the SSV

Narrowing of the joint gap was associated with a higher Franklin radiolucency grade (β = −0.14, *p* < 0.001, Table 4) and trended toward lower SSV (β = 1.39, *p* = 0.06). A decrease in the lateral humeral offset also showed a tendency toward a higher radiolucency grade (β = −0.06, *p* = 0.09). No significant association was observed for the change in acromiohumeral distance.

### 3.6. Sensitivity Analysis

We did not see any correlation between preoperative osteoarthritic glenoid changes (Walch classification) and loosening of the glenoid component, despite having a high number of B glenoids (B1 = 13%, B2 = 18%, B3 = 7%). Interestingly, women showed significantly higher grades in radiolucency compared to men (β = −0.97, *p* < 0.001, Table 5). No difference was found between low and high ASA classifications (American Society of Anesthesiologists) and the glenoid component sizes.

## 4. Discussion

The main reason for revision surgery in aTSA is aseptic loosening of the glenoid component [15,21,32,33]. Recently, new glenoid component designs (e.g., backside design, method of fixation, implant material) showed promising short-term results [34,35]. Despite these reports, we detected early radiographic signs of implant loosening with superior and medial shift of the humeral head and high grades of radiolucency after two years. Furthermore, these radiographic parameters correlated with decreased patient-reported outcomes.

Although a certain amount of radiolucent lines and radiologic osteolysis correlates with loosening of the PE implant, they do not always lead to decreased functional and clinical outcomes. According to Torchia et al., there is a critical threshold of radiolucency (over >1.5 mm) that leads to pain [4]. Moreover, symptoms can be absent, and deterioration of movement does not perfectly correlate with the magnitude of radiological loosening signs [9,36,37,38].

The limited reliability of determining radiolucency around the implant with plain radiographies is known, but routine CT scans during the follow-up can expose the patient to high doses of radiation, and are not logistically or economically feasible. In this context, the described parameters of medialization and cranialization might provide a cost-effective alternative.

The concept of a “floating glenoid” was introduced by investigating glenoid components of patients with long-term follow-ups after aTSA (15 to 21 years) [39]. Although there was a major lysis of the glenoid, patients had little to no functional limitations. One explanation is that as long as the expansion of the lysis does not involve cortical bone, there is no major destabilization of the implant and therefore no pain [40]. This would confirm our findings. Both the constant score and a patient-reported outcome (SSV) in our population decreased between two and five years follow-up after showing continuously improving scores for the first two years postoperatively. Looking at the current literature, most studies on mid-term stemless aTSA outline a comparison between preoperative and postoperative status, without describing performance over time at several follow-up points [41]. One explanation for worsening outcome scores after two years in our population may be that, in a relevant number of cases, a high grade of loosening caused functional deterioration and pain. Whether this sharp functional decline is implant-related warrants a materiovigilance investigation. We observed lowering of the subjective shoulder value, a stated and established measure for the clinical outcome of shoulder function [25], only with gross loosening (grade 5 according to Lazarus [19]). A previous investigation of a long term follow-up (nine years) of a keeled fixation of the same implant we used in our study showed complete radiolucency around the implant in two patients out of four [22]. Kilian et al. compared the five-year outcome of pegged and keeled cemented PE glenoids in 38 patients and found overall significantly worse outcome scores (ASES—American shoulder and elbow surgeons—score, constant score, WOOS—Western Ontario Osteoarthritis of the Shoulder—index, SANE—Single Assessment Numeric Evaluation), forward flexion, and abduction in patients with grade 4 and 5 loosening, but no difference between the two cohorts [42]. We did not see an association between grades of radiolucent lines below 5 and clinical outcome parameters across time points. One can speculate whether a correlation could have been detected with a more sensitive radiologic tool. Yian et al. were able to demonstrate a significant association between radiolucent lines and pain with CT scans, but not with conventional radiographs [43].

Since radiolucent lines are not always clinically relevant, it is of interest to find additional radiographic parameters associated with gross loosening, implant subsidence, and implant failure. In our patients, parameters that address cranialization of the humeral head (AHD) and medialization (JG and LHO) correlated with worse outcome scores of SSV and radiolucency. Another possible factor that could be correlated with later loosening might be the glenoid preparation and fixation technique. Dillian et al. investigated the revision risk for various glenoid components using as an all-cemented PE glenoid representative the same implant we used. They observed in their cohort of over 5000 primary elective aTSAs a higher risk for revision due to glenoid loosening with PE all-cemented pegged and keeled glenoids compared to metal hybrid and central-pegged ingrowth glenoid implants [44]. Collins et al. investigated the effect of different methods of preparation of the glenoid bone on the displacement and deformation of a glenoid component under eccentric loading in a cadaver study. The study showed that hand burring was associated with less displacement and deformation than simple removal of cartilage with a curette. Substantial posterior bone deficiency of the reamed glenoid was not associated with significant increases of displacement and deformation [18]. Walch et al. observed three specific correlations in their retrospective multicenter investigation on patterns of loosening of 518 patients with PE keeled glenoid components and a minimum of five years follow-up. Inferior glenoid positioning, superior tilt immediately postoperatively, and superior subluxation of the humeral head were all correlated with superior tilting of the implant (in 10% of the patients). A subsidence of the glenoid PE was observed with reaming (7.9%), and posterior tilting was observed with B-glenoids and excessive reaming (6.4%) [33]. Little data exists in anatomic TSA regarding the association of radiolucency with cemented glenoid implant loosening and gender. Fox et al. and Schoch et al. reported no differences regarding the occurrence of radiolucent lines on the side of the glenoid implant (cemented keeled or pegged) between women and men [45,46], while we observed significantly higher RLL grades in women. It would be interesting to further analyze this association regarding the aspect of e.g., the bone density parameter in a future project. Fox et al. supports our result that glenoid size is not a risk factor for radiolucent lines around the implant [45].

For determining subsidence and/or wear of the PE implant, we introduced the measurement of the JG. In our study, a correlation between joint gap width and loosening was indeed seen, which can be defined as an evidence for subsidence. A decrease in LHO correlated with loosening as well. The bony landmarks for our measurement of the LHO can be detected with accuracy and reproducibility in plain radiographies [47,48]. Although we did not see a recognizable single pattern of loosening of the PE, first signs of progression of radiolucency and loosening occurred mostly in the inferior part of the glenoid [9,37,49].

**Figure 4:**
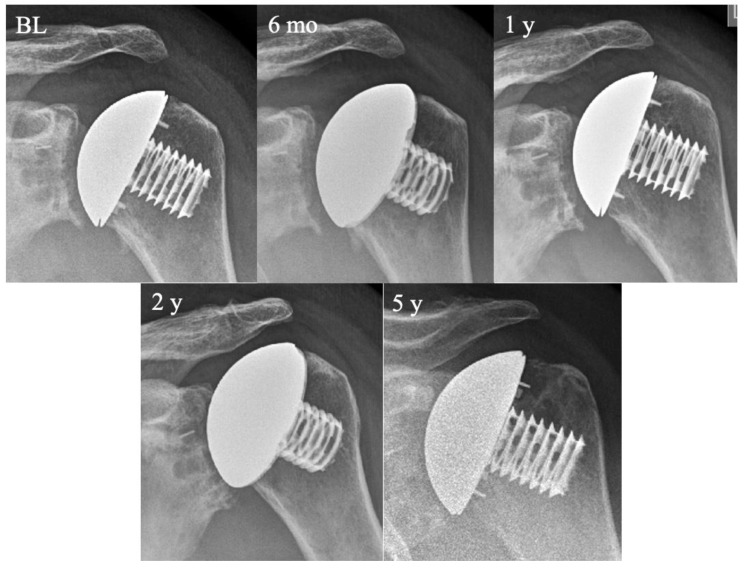
Progression of medialization and cranialization over time. Anteroposterior radiographs with the patient positioned in internal rotation. Images depict time points immediately after surgery (baseline, BL) and follow-ups after six months (6 mo) and one, two, and five years later (from top left to bottom right).

Our primary aim was to find predictive radiologic parameters for loosening. The same radiologic parameters correlating with loosening (LHO and JG) also showed a correlation with the subjective shoulder value, confirming their applicability as screening parameters (Figure 4).

Limitations of our study include a reduced number of patients with a five-year follow-up, which is expected in an observational cohort that is based on clinical routine [50]. Furthermore, since patients were included until end of 2016, not all of the five-year examinations could be carried out at the time point of submission. Nevertheless, our study was sufficiently powered to detect clinically meaningful associations. Additionally, our first post-operative baseline was only at six months. Another possible limitation is that we did not perform standard follow-up sonographic examination to confirm cuff integrity. A decrease of AHD could be mainly caused either by cuff insufficiency or by glenoid implant subsidence, causing coronal joint instability and cranialization of the humeral head. We also did not assess retro- or anteversion of the glenoid, since CT imaging was not performed routinely. Therefore, we cannot address this parameter, which may play a role in glenoid implant loosening [40]. Strengths of this study are that we analyzed one single implant model, same postoperative rehabilitation therapy was consistently performed by all patients, the same investigator did all radiologic measurements, minimizing inter-observer variability, and surgery was performed predominantly by two experienced senior surgeons. Having more than one surgeon also minimizes the potential bias that our findings are based predominantly on the surgical technique (e.g., reaming of the glenoid and cementing technique).

## 5. Conclusions

Radiological parameters that reflect medialization and cranialization after an anatomical total shoulder prosthesis with a cemented pegged glenoid are useful predictors of a worse outcome and possible risk of future clinical failure of the glenoid component due to implant loosening.

## Figures and Tables

**Table 1:** Baseline characteristics.

	N (%)	Mean (SD)
Age	150	67 (8)
Age category		
≤60	30 (20)	
61–70	66 (44)	
>70	54 (36)	
Gender		
Women	88 (59)	
Men	62 (41)	
Smoking		
No	119 (92)	
Yes	10 (8)	
BMI	68	28 (6)
ASA Physical Status Classification System		
I. Healthy patient	18 (12)	
II. Mild systemic disease	78 (52)	
III. Severe systemic disease	52 (35)	
IV. Severe systemic disease that is a constant threat to life	1 (1)	
Pre-operative pain (0–10)	135	6 (2)
Constant score (0–100)	130	36 (16)
Subjective Shoulder Value (0–100) *	67	44 (23)
Abduction °	135	79 (28)
Flexion °	135	96 (28)
Abduction strength, affected arm (kg)	133	1 (3)
Abduction strength, unaffected arm (kg)	127	7 (3)
EQ-VAS (0–100) *	72	68 (19)
Walch classification		
A1	30 (20)	
A2	56 (38)	
B1	19 (13)	
B2	26 (18)	
B3	10 (7)	
C	6 (4)	
Pegged glenoid component size		
Small	87 (58)	
Medium	50 (33)	
Large	13 (9)	
Humeral Head component size		
39/16	7 (5)	
41/16	27 (18)	
43/16	36 (24)	
45/17	31 (21)	
47/18	22 (15)	
49/18	16 (11)	
51/19	8 (5)	
53/20	3 (2)	

Abbreviations: ASA, American Society of Anesthesiologists; BMI, body mass index; SD, standard deviation; VAS, visual analogue scale; Walch classification: A1, minor central erosion; A2, severe or major central erosion; B1, no obvious glenoid erosion with posterior joint space narrowing, subchondral sclerosis and osteophytes; B2, obvious erosions of posterior glenoid with biconcave appearance; B3, uniconcavity, medialization, retroversion and subluxation; C, glenoid retroversion > 25°. Active ranges of motion are presented. * Parameter introduced in 2014.

**Table 2:** Change in radiographic parameters with time.

Radiographic Parameter	Six Months	One Year	Two Years	Five Years	*p*-Value (Two Years vs. Six Months)	*p*-Value (Five Years vs. Six Months)
Acromiohumeral distance (mm)	12.7 (0.3)	12.5 (0.3)	12.1 (0.3)	10.4 (0.4)	0.17	<0.001
Lateral humeral offset (mm)	52 (0.3)	51.9 (0.3)	51.5 (0.3)	50.5 (0.4)	0.02	<0.001
Joint gap width (mm)	2.9 (0.1)	2.8 (0.1)	2.6 (0.1)	1.4 (0.2)	0.17	<0.001
Franklin radiolucency grade	2.2 (0.1)	2.7 (0.1)	3.1 (0.1)	3.8 (0.1)	<0.001	<0.001

Adjusted means are presented. Standard errors are in parentheses.

**Table 3:** Association between radiographic parameters and constant score and subjective shoulder value across time points.

Radiographic Parameter	Constant Score		Subjective Shoulder Value	
	Beta (SE)	*p*-Value	Beta (SE)	*p*-Value
Acromiohumeral distance	0.03 (0.20)	0.90	0.50 (0.27)	0.07
Lateral humeral offset	0.27 (0.23)	0.23	0.34 (0.35)	0.34
Joint gap width	0.04 (0.45)	0.92	1.51 (0.70)	0.03
Franklin grade ≤ 1 (Ref.)				
Franklin grade 2	1.58 (1.94)	0.42	1.45 (2.92)	0.62
Franklin grade 3	0.39 (1.99)	0.85	0.70 (3.13)	0.82
Franklin grade 4	0.27 (2.29)	0.90	−1.55 (3.62)	0.67
Franklin grade 5	−0.32 (3.27)	0.92	−14.48 (4.84)	<0.01

Regression coefficients from a mixed-model linear regression are presented. Standard errors are in parentheses.

**Table 4:** Association between change in radiographic parameters (Δ from six months) and Franklin radiolucency grade, constant score, and subjective shoulder value (at one, two, and five years).

Change in Radiographic Parameter	Franklin Grade		CS		SSV	
	Beta (SE)	*p*-Value	Beta (SE)	*p*-Value	Beta (SE)	*p*-Value
Δ Acromiohumeral distance	−0.02 (0.02)	0.33	−0.3 (0.29)	0.30	0.49 (0.37)	0.18
Δ Lateral humeral offset	−0.06 (0.03)	0.09	0.48 (0.43)	0.27	0.76 (0.65)	0.24
Δ Joint gap width	−0.14 (0.04)	<0.001	0.39 (0.54)	0.47	1.39 (0.75)	0.06

Regression coefficients from a mixed-model linear regression are presented. Standard errors are in parentheses. Δ stands for the difference from six months over time respective to the different parameters.

**Table 5:** Association between gender, ASA class, glenoid component size, and the Franklin radiolucency grade (dependent variable).

Variable	Beta (SE)	*p*-Value
Women (Ref.)		
Men	−0.97 (0.2)	<0.001
ASA class 1 and 2 (Ref.)		
ASA class 3 and 4	0.02 (0.2)	0.9
Glenoid component: Small (Ref.)		
Glenoid component: Medium	0.33 (0.2)	0.1
Glenoid component: Large	0.2 (0.3)	0.6

Regression coefficients from a mixed-model linear regression are presented. Standard errors are in parentheses. Model was adjusted for age and timepoint. Abbreviations: ASA, American Society of Anesthesiologists.

## Data Availability

The Schulthess Shoulder Arthroplasty Registry data availability policy and procedures are outlined in Marzel et al.
